# The regulatory role of DPP4 in atherosclerotic disease

**DOI:** 10.1186/s12933-017-0558-y

**Published:** 2017-06-15

**Authors:** Lihua Duan, Xiaoquan Rao, Chang Xia, Sanjay Rajagopalan, Jixin Zhong

**Affiliations:** 1 Department of Rheumatology and Clinical Immunology, The First Affiliated Hospital of Xiamen University, Xiamen, 361003 Fujian China; 2 Cardiovascular Research Institute, School of Medicine, Case Western Reserve University, 2103 Cornell Rd., Wolstein Research Building 4525, Cleveland, OH 44106 USA; 3 Department of Microbiology and Immunology, Wuhan Polytechnic University, Wuhan, 430023 Hubei China

**Keywords:** Dipeptidyl peptidase, Gliptins, Atherosclerosis, Incretin, DPP4 inhibitor

## Abstract

The increasing prevalence of atherosclerosis has become a worldwide health concern. Although significant progress has been made in the understanding of atherosclerosis pathogenesis, the underlying mechanisms are not fully understood. Recent studies suggest dipeptidyl peptidase-4 (DPP4), a regulator of inflammation and metabolism, may be involved in the development of atherosclerotic diseases. There has been increasing clinical and pre-clinical evidence showing DPP4-incretin axis is involved in cardiovascular disease. Although the cardiovascular outcome of DPP4 inhibition or incretin analogues has been or being evaluated by several large scale clinical trials, the exact role of DPP4 in atherosclerotic diseases is not completely understood. In the current review, we will summarize the recent advances in direct and indirect regulatory role of DPP4 in atherosclerosis.

## Background

Atherosclerosis, the primary cause of cardiovascular disease, is a chronic progressive condition with lipids and fibrous elements accumulated in the vessel wall of large arteries [1–3]. It accounts for 50% of all deaths in the Western countries [4]. The increasing prevalence of atherosclerosis has become a worldwide health concern [5]. Despite of rapid progress in atherosclerosis research in the recent years, the underlying mechanisms remain elusive. Dipeptidyl peptidase-4 (DPP4) inhibitors are a novel class of glucose-lowering drugs [6]. Various studies suggest DPP4 inhibitors may also possess cardioprotective effect [7–12], including atherosclerosis [13–15]. In the current review, we will summarize the recent advances in direct and indirect regulatory role of DPP4 in cardiovascular disease, especially in atherosclerosis.

## Experimental evidence of the beneficial effects of DDP4 inhibition on cardiovascular disease

Dipeptidyl peptidase-4 is a membrane-bound enzyme that cleaves N-terminal dipeptide from its substrates. It belongs to the S9b dipeptidyl peptidases family [16]. There are over a dozen proteins identified as the substrates of DPP4. These include glucagon-like peptide (GLP) -1 and -2, glucose-dependent insulinotropic peptide (GIP) [17], stromal-cell-derived factor-1 (SDF-1) [18], GM-CSF [19], regulated on activation normal T-cell expressed and presumably secreted (RANTES) [20], eotaxin [21], neuropeptide Y (NPY) [22], etc. The molecular basis of catalytic activity of DPP4 has been reviewed elsewhere and we will only discuss the recent advances in DPP4 biology [23–25].

DPP4 inhibitors are being increasingly used in clinical for the treatment of type 2 diabetes as they are safe and weight neutral [26–32]. They preserve incretin hormones such as GLP-1 and GIP by inhibiting DPP4-mediated degradation, and thus could promote postprandial insulin secretion and reduce pancreatic β cell apoptosis [33–36]. Furthermore, antiapoptotic effects of DPP-4 inhibitor were observed in human umbilical vein endothelial cells (HUVECs) cultured under hypoxic condition. Besides, the CXCR4 antagonist or Stat3 inhibitor can abolish this effect. These results suggested that DPP-4 inhibitor has a potential for protecting vessels where CXCR4/Stat3 pathways might be involved [37]. In animal experiments, Salim HM et al. demonstrated that linagliptin ameliorated atherogenesis in non-diabetic *ApoE* ^−*/*−^ mice by inhibiting the oxidative stress [38], and it also showed that linagliptin prevents the development of aortic and endothelial stiffness in female mice resulting from western diet-induced vascular abnormalities [39]. We have previously also shown long-term enzymatic inhibition of DPP4 by sitagliptin or alogliptin reduced atherosclerotic plaque burden in *Ldlr* ^−*/*−^ and *ApoE* ^−*/*−^ mice, accompanied by reduced monocyte activation and migration [40]. In consistency with that, a recent study demonstrated 12-week anagliptin treatment suppressed atherosclerosis progression and macrophage infiltration in the plaque in cholesterol-fed rabbits [41], and 20-week teneligliptin administration inhibited the development of atherosclerosis in the aortic arch in *ApoE* ^−*/*−^ mice through restraining macrophage infiltration, downregulating lipid deposition and MCP-1 expression, and in vitro experiment GLP-1 analogue treatment suppressed the pro-inflammatory cytokines production [42]. Not only by reducing monocyte activation and migration, DPP4 inhibitor sitagliptin can attenuate atherosclerosis by promoting M2 macrophages polarization [43]. Acute in vitro DPP4 inhibition also relaxed pre-constricted aorta segments by activating Src-Akt-eNOS pathway in a GLP-1-independent manner [44]. In addition, combination therapy with DPP4 inhibitor and sodium-glucoase cotransporter 2 inhibitor showed the greatest suppression of plaque volume in the aortic root of diabetic *ApoE* ^−*/*−^ mice [45].

Interestingly, DPP4 inhibitor but not GLP-1 or GIP reduced incidence of angiotensin II-induced abdominal aortic aneurysm in *ApoE* ^−*/*−^ mice (40% in DPP4 inhibition group vs. 70% in control group) [46]. Using an experimental myocardial infarction model, Sauvé et al. examined the effects of DPP4 enzymatic inhibition or genetic deletion of DPP4 on cardiovascular function in normoglycemic and diabetic mice [47]. *Dpp4* ^−*/*−^ mice displayed normal cardiac structure and function, with increased levels of phosphorylated AKT (pAKT), pGSK3β, and atrial natriuretic peptide (ANP) in the heart. After left anterior descending (LAD) coronary artery ligation, *Dpp4* ^−*/*−^ mice showed a modestly improved survival and functional recovery from ischemia–reperfusion injury as measured by left ventricle developed pressure. Treatment of DPP4 enzymatic inhibitor sitagliptin also reduced mortality and enhanced functional recovery from ischemia–reperfusion injury in wild-type diabetic mice after myocardial infarction [47]. Consistently, treatment with DPP-4 inhibitor vildagliptin markedly improved the survival rate after myocardial infarction in type 2 diabetic rats, and the protective effect was linked with restoration of autophagic response resulting from increased both LC3-II protein level and autophagosomes after vildagliptin treatment [48]. Furthermore, Ma et al. also DPP4 inhibition exerted a potential strategy for treating cerebral vascular complications in T2DM through reducing oxidative stress and suppressing blood–brain barrier disruption [49]. Although a number of animal studies suggest DPP4 inhibition may have cardiovascular beneficial effects in addition to its glycemic lowering effect [8, 50, 51], while there was no study uncover the precise cells and tissues which is critical for incretin degradation. Recently, a study clearly showed that levels of soluble plasma DPP4 activity, incretin degradation, and glucose regulation rely heavily on endothelial cell-derived DPP4. Surprisingly, fasting GIP, but not GLP-1, was mainly degraded by bone marrow-derived DPP4 [52], and a study also demonstrated that active GIP levels were increased more than GLP-1 after DPP-4 inhibitor treatment [53]. Shigeta and colleagues also reported DPP4 expressed on the cardiac capillary endothelia is up-regulated by diabetes, resulting in a reduction in myocardial SDF-1 and increased interstitial fibrosis. Both genetic and pharmacological inhibition of DPP4 restored diabetes-induced SDF-1 and reversed pressure overload-induced diastolic left ventricular dysfunction by GLP-1-dependent mechanisms [54].

## Clinical trials on the cardiovascular effects of DPP4 inhibition

In contrast to the consistent beneficial cardiovascular effects of DPP4 inhibition on experimental animal models, the cardiovascular effects of DPP4 in human studies on seem more complicated and inconsistent (Table 1) [55–63].

**Table 1:** Dipeptidyl peptidase 4 inhibitors and atherosclerosis outcome trials

Study	Study design	Drug	Duration	Result
SPIKE	Randomized/blinded-endpoint	Sitagliptin	104-week	The mean IMT and left maximum IMT of common carotid arteries were reduced
SPEAD-A	Randomized/blinded-end point	Alogliptin	24-month	The progression of carotid IMT was attenuated
RELEASE	Randomized/double-blind	Linagliptin	26 weeks	Aortic PWV was decreased
PROLOGUE	Randomized/blinded-end point	Sitagliptin	24-month	No additional effect on the progression of carotid IMT
TRACT	Non-randomized	Alogliptin	48-week	Not end. Intermediate coronary artery stenosis of T2DM patients will be evaluated by CCTA

CCTA, coronary computed tomography angiography; IMT, intima-media thickness; PWV, pulse wave velocity; T2DM, type 2 diabetes mellitus

Three months of vildagliptin (50 mg twice daily) or sitagliptin (100 mg once daily) also significantly reduced carotid intima-media thickness (IMT), a surrogate marker of atherosclerosis, in patients with type 2 diabetes [64]. Further analysis indicated this maybe relates to the reduction in daily acute glucose fluctuation and plasma LDL level [64]. A randomized controlled trial, the sitagliptin preventive study of IMT evaluation (SPIKE), demonstrated that sitagliptin 104-week treatment had a greater changes in the mean and left maximum IMT of the common carotid arteries, which measured by echography [65]. Besides, a study of preventive effects of alogliptin on diabetic atherosclerosis (SPEAD-A), which includes T2DM patients with alogliptin treatment (n = 172) or conventional treatment (n = 169), showed that 20 months of alogliptin treatment significantly changed the mean common and the right and left maximum IMT of the carotid arteries (P = 0.022, P = 0.025, P = 0.013, respectively) [66]. Similarly, in insulin-treated patients with type 2 diabetes mellitus (T2DM), a recently randomized trial was analyzed in according with SPIKE study, and the results showed that sitagliptin treatment could regress carotid IMT. The percentage of patients who showed a decrease of ≥0.10 mm in mean-IMT-CCA in the sitagliptin group was 28.9%, while the conventional group the percentage was 16.4% (P  =  0.022) [67]. Furthermore, aortic pulse wave velocity, a marker of early atherosclerosis [68], were significantly decreased in T2DM patients with 26 weeks of linagliptin administration in a small study with a limited number of participants (RELEASE) when compared with placebo (P  =  0.035) [69]. Kutoh et al. [70] recently reported that 12.5–25 mg/day alogliptin monotherapy for 3 months significantly improved insulin resistance and beta cell function and reduced atherogic lipids in type 2 diabetic patients with a better glycemic response to alogliptin. In another study, however, DPP4 inhibitor sitagliptin as an add-on in type 2 diabetic patients for 6 months did not improve atherogenic lipids including total cholesterol, LDL cholesterol, and malondialdehyde-modified LDL (an oxidized LDL increased in diabetes) [71]. In addition to previous study, a multicenter Randomized Controlled Trial (prospective, randomized, open label, blinded endpoint, PROLOGUE design) also revealed that sitagliptin treatment had no effect on the progression of carotid IMT in T2DM patients [72]. A further sub-analysis of the PROLOGUE study also showed that no significant effect of sitagliptin on the arterial stiffness and endothelial function in T2DM patients which are thought to contribute to the occurrence of atherosclerosis [73, 74]. However, PROLOGUE randomized controlled trial used carotid IMT as a marker of atherosclerosis, which can not entirely represent the status of atherosclerosis. Regarding the different conclusion reported by different clinical trials, Nozue et al. [75] design a prospective, non-randomized, multicenter trial performed in Japan, where T2DM patients who have intermediate coronary artery stenosis will be recruited. The diameter stenosis will be evaluated by coronary computed tomography angiography (CCTA) at the starting point and ending point (48 weeks alogliptin treatment). This clinical trial will provide a strong evidence for the effect of alogliptin in the anti-atherosclerosis.

It is reported that treatment with vildagliptin for 4 weeks in patients with type 2 diabetes improved forearm blood vasodilator responses to intra-arterially administered acetylcholine, but not to sodium nitroprusside [76]. Nakamura et al. [77] also reported that sitagliptin treatment for 12 weeks significantly improved flow-mediated dilatation in diabetic patients uncontrolled on sulfonylurea, metformin or pioglitazone treatment. In contrast, another study demonstrated that the flow-mediated dilation was suppressed by both sitagliptin and alogliptin [78].

Several large scale clinical trials were carried out to evaluate the cardiovascular effects of DPP4 inhibitors and GLP-1R agonists [79–83]. The cardiovascular safety of DPP4 inhibitors, especially in heart failure, has been questioned since 2013 when the first 2 large clinical trials assessing the cardiovascular safety of DPP4 inhibitors (EXAMINE and SAVOR-TIMI 53) were completed [84, 85]. Both of these trials demonstrated that DPP4 inhibitors do not increase overall cardiovascular risks. However, there was also no beneficial effect on primary cardiovascular outcomes in both trials. Interestingly, the SAVOR-TIMI 53 trial reported a 27% increase in hospitalization for heart failure in saxagliptin group, although no increased heart failure-related death was observed (3.5% vs. 2.8%; hazard ratio, 95% CI 1.27, 1.07–1.51; P = 0.007) [85]. In the EXAMINE trial, there were no heart failure-related outcomes reported in their first report although there were 28% of subjects having congestive heart failure at baseline [84]. After re-analysis, the heart failure hospitalization data of the EXAMINE trial was published in 2015, reporting a negative effect of alogliptin on the occurrences of heart failure hospitalization (3.1% in alogliptin vs. 2.9% in placebo group; HR 1.07, 0.79–1.46; P = 0.68) [86]. The 3rd large scale trial assessing the cardiovascular effect of DPP4 inhibitor, TECOS, finished in 2015. In consistency with the first two trials, sitagliptin neither increases nor reduces cardiovascular events (primary outcome incidence, 11.4% vs. 11.6%; HR 0.98, 95% CI 0.88–1.09) [87]. More importantly, the hospitalization rate for heart failure was identical in sitagliptin and placebo (3.1% vs. 3.1%; HR 1.00, 0.83–1.20; P = 0.98). Furthermore, a pre-specified patient-level pooled analysis of all available double-blind, randomized, controlled trials, ≥ 12 weeks’ duration (19 trials, 9459 subjects) also demonstrated that linagliptin was not associated with increased cardiovascular risk in T2DM patients [88]. Based on the three trials currently available, it seems the excessive heart failure effect may not be a class effect of DPP4 inhibitors. This will be further tested by the ongoing trials on linagliptin (CAROLINA and CARMELINA), both of which are expected to be completed in 2018.

There were two large trials examining the cardiovascular safety of GLP-1R agonists recently completed, one on lixisenatide (ELIXA) and one on liraglutide (LEADER). The ELIXA trial indicates lixisenatide is neither inferior nor superior to placebo in terms of cardiovascular safety. In addition, there were similar hospitalization rates for heart failure compared with placebo (HR 0.96; 95% CI 0.75–1.23) [89]. Surprisingly, the LEADER trial indicates a beneficial effect of liraglutide on cardiovascular outcomes. Liraglutide significantly reduced the risk of major adverse cardiovascular events [90]. These data suggest the heart failure effect of saxagliptin is independent of preservation of incretins.

## Mechanisms by which DDP4 participates in cardiovascular disease

Chronic inflammation is key process in the pathogenesis of atherosclerosis. DPP4 inhibition may reduce monocyte migration to atherosclerotic plaque in response to TNFα and soluble DPP4 [40], it also up-regulates the adiponectin expression which exerts anti-inflammation [91]. Recent study also showed that T2DM patients with GLP-1 analogues or DPP4 inhibitors treatment had a significant smaller portion of plaque area occupied by macrophages and T-cells compared with the patients who never used GLP-1 analogues or DPP4 inhibitors, and the difference was closely associated with adiponectin and adaptor protein PH domain and leucine zipper containing 1 (APPL1) which can be induced by GLP-1 in ex vivo cell culture system [92]. Inhibition of DPP4 also results in elevation of SDF-1, a chemoattractant for many types of hematopoietic stem cells and progenitor cells such as cardiac stem cells, endothelial progenitor cells, and mesenchymal stem cells [18, 93–96]. Enhancement of SDF-1-mediated hematopoietic stem cell and progenitor cell chemotaxisand repopulation may then promote neovascularization and recovery of tissue injury [18]. Nevertheless, the enhancement of angiogenesis through SDF-1-mediated epithelial progenitor cell migration and repopulation may contribute to plaque instability [97].

Many groups have shown that DPP4 inhibition is able to lower lipid levels [98–103]. DPP4 inhibitors treatment following a high-fat liquid formula significantly reduced triglyceride-rich lipoproteins apoB48 in healthy individuals [103–105]. Three-month alogliptin monotherapy has been shown to reduce atherogic lipids in type 2 diabetic patients who had a better glycemic response to alogliptin [70]. In consistency, diabetic patients treated with vildagliptin (50 mg bid for 4 weeks) [106] or sitagliptin (100 mg/day for >6 weeks) [107] or alogliptin (25 mg/day for 7 days) [108] also displayed decreased postprandial plasma triglyceride, chylomicron apoB48 after mixed meal challenge.

In a follow-up study of SAVOR-TIMI 53, the authors suggest the increased heart failure hospitalization rate in saxagliptin group might be related to previous history of heart failure, low glomerular filtrate rate (eGFR < 60 ml/m), highbrain natriuretic peptide (BNP) and high albumin/creatinine ratio. In addition, Zhou et al. [109] presented results that DPP4 inhibitor can evoke significant diuretic and natriuretic responses and increased GFR by potentially affecting on renal sodium and water handling, which might be benefit to heart failure. BNP is able to promote vasodilation and natriuresis and it can be degraded by DPP4 to a less potent metabolite BNP(3–32) [110]. BNP also promotes norepinephrine release, which may counteract the beneficial effects of BNP-mediated vasodilation and natriuresis on heart failure [111]. Treatment of sitagliptin 200 mg/day increased forearm blood flow and decreased forearm vascular resistance in healthy humans, while BNP infusion also caused vasodilation in a dose-dependent manner [112, 113]. However, the authors did not observe an effect of sitagliptin on BNP-mediated vasodilation [112]. In addition, DPP4 inhibition has also been shown to induce vasodilation by promoting endothelial nitric oxide synthase (eNOS)-mediated NO release independent of GLP-1R signaling [44, 114]. DPP4 inhibition-mediated GLP-1R signaling may also activate Epac2 and induce atrial natriuretic peptide (ANP) release from the atrium, and thus dilate vessels [115]. DPP4 inhibition has been shown to increase sympathetic activation by preserving substance P [113] and thus may increase systolic blood pressure [112]. GLP-1R agonist may also activate sympathetic nervous system and increase heart rate [116]. A recent study reported that genetic deficiency of DPP4 improve cardiac function, whereas chemical inhibition of DPP4 by MK-0626 induced cardiac hypertrophy and impaired cardiac function, indicating drug related unspecific effects might negatively impact cardiac function [117].

## Conclusions

**Fig. 1:**
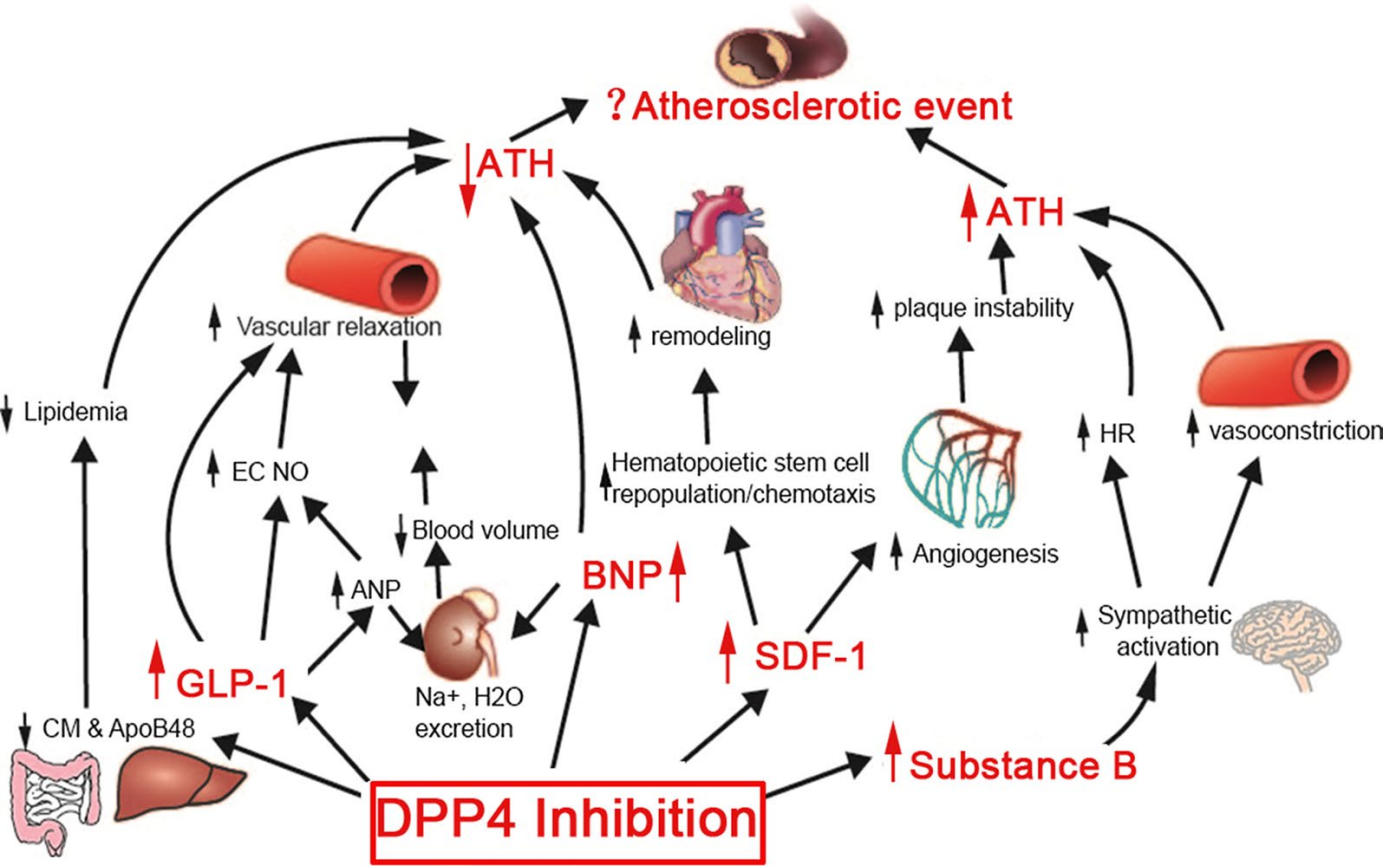
Mechanisms underlying the cardiovascular action of DPP4 inhibition: ATH, atherosclerosis; ANP, atrial natriuretic peptide; CM, chylomicron; EC, endothelial cell; HR, heart rate; NO, nitric oxide; SDF-1, stromal-cell-derived factor-1

The cardiovascular side effect is a big concern of oral anti-diabetic drugs. The cardiovascular safety of DPP4 inhibitors, as a new class of oral anti-diabetic drugs, has drawn much attention. The three large trials recently completed indicate DPP4 inhibitors as a class are safe from cardiovascular perspective. Many preclinical and clinical studies have shown DPP4 inhibitors may modulate atherosclerotic disease by reducing plasma lipids, suppressing inflammation, and promoting vascular relaxation (Fig. 1). However, inhibition of DPP4 may also exacerbate cardiovascular disease by enhancing sympathetic activation and angiogenesis. Ongoing trials assessing the cardiovascular effects of DPP4 inhibitors and GLP-1R agonists will provide further insights into the cardiovascular actions of DPP4 inhibitors.

## Abbreviations

DPP4: dipeptidyl peptidase-4
GLP-1: glucagon-like peptide-1
GIP: glucose-dependent insulinotropic peptide
SDF-1: stromal-cell-derived factor-1
RANTES: regulated on activation normal T cell expressed and presumably secreted
NPY: neuropeptide
